# Giant Lipoma: A Case Report

**DOI:** 10.7759/cureus.53000

**Published:** 2024-01-26

**Authors:** Chinmay Kher, Swarupa Chakole

**Affiliations:** 1 Surgery, Datta Meghe Institute of Medical Sciences, Wardha, IND; 2 Community Medicine, Datta Meghe Institute of Medical Sciences, Wardha, IND

**Keywords:** liposuction, recurrent lipoma, liposarcoma, adipose tissue, benign tumor, deformity, giant lipoma, lipoma

## Abstract

Lipomas are one of the most common, benign, slow-growing tumours composed of adipose (fat) tissue. These soft, rubbery lumps are usually painless and move easily when touched. Lipomas are generally small, ranging from less than an inch to a few inches in diameter. However, when a lipoma grows to a size larger than 10 cm (about 4 in), it is referred to as a giant lipoma. Only about 1% of all lipomas can be called "giant". Though usually they are benign, in the case of a very large lipoma it is essential to rule out the possibility of malignancy before embarking on its surgical treatment. Lipomas can occur anywhere in the body, but they are most commonly found on the neck, shoulders, back, abdomen, arms, or thighs. Here, we present a case of a 42-year-old woman with a giant lipoma over her left scapula.

## Introduction

A lipoma is a benign, slow-growing tumour composed of adipocytes. These soft, rubbery lumps are usually painless and easily move when touched. Lipomas are among the most common types of soft tissue tumours, with a prevalence of 2.1 per 1000 [1]. Giant lipomas, however, account for only 1% of all lipomas. While they can occur anywhere in the body, they are most commonly found on the neck, shoulders, back, abdomen, arms, or thighs [2]. Lipomas are generally small, ranging from less than an inch to a few inches in diameter. However, when a lipoma grows to a size larger than 10 cm (about 4 in), it might be referred to as a giant lipoma [3,4]. Such giant lipomas cause multiple difficulties to the patients, like physical deformities, pain, difficulty in sleeping, and compressions of nerves or vital structures [4].

## Case presentation

**Figure 1:**
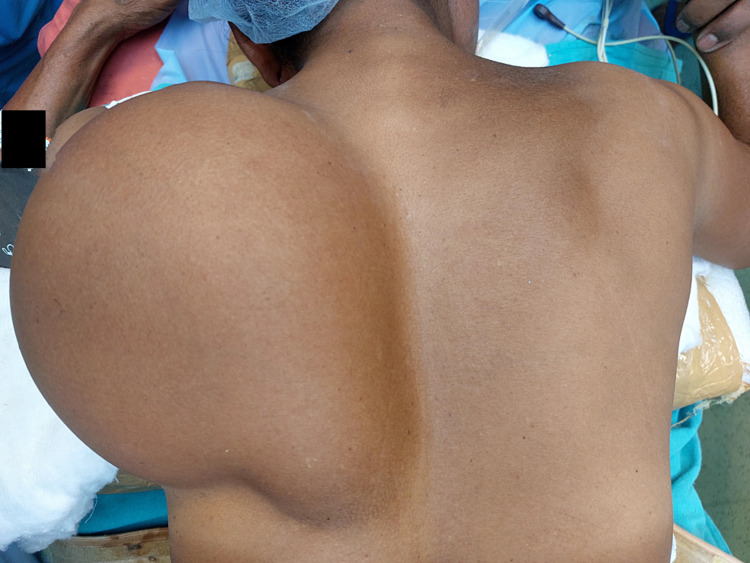
Image of the patient's back at the time of presentation A swelling of 10 cm × 12 cm x 7 cm was present over the left scapula.

A 42-year-old lady presented to the surgery clinic with complaints of swelling on the left side of her back, which was 10 cm x 12 cm x 7 cm in size on examination. The patient gave a history of detecting the swelling when it was the size of a small lemon (approximately 2 x 2 cm) and progressed gradually to its present size over the last three years. The swelling was always painless and not associated with any other symptom. There was no history of trauma. The patient had no functional constraints, such as carrying out day-to-day activities or postural disturbances. The patient felt embarrassed to meet people due to her physical deformity and worried about the gradual growth in the size of the tumour. On physical examination, the swelling was well-circumscribed, was freely movable and its margin slipped under the palpating fingers (Figure 1).

**Figure 2:**
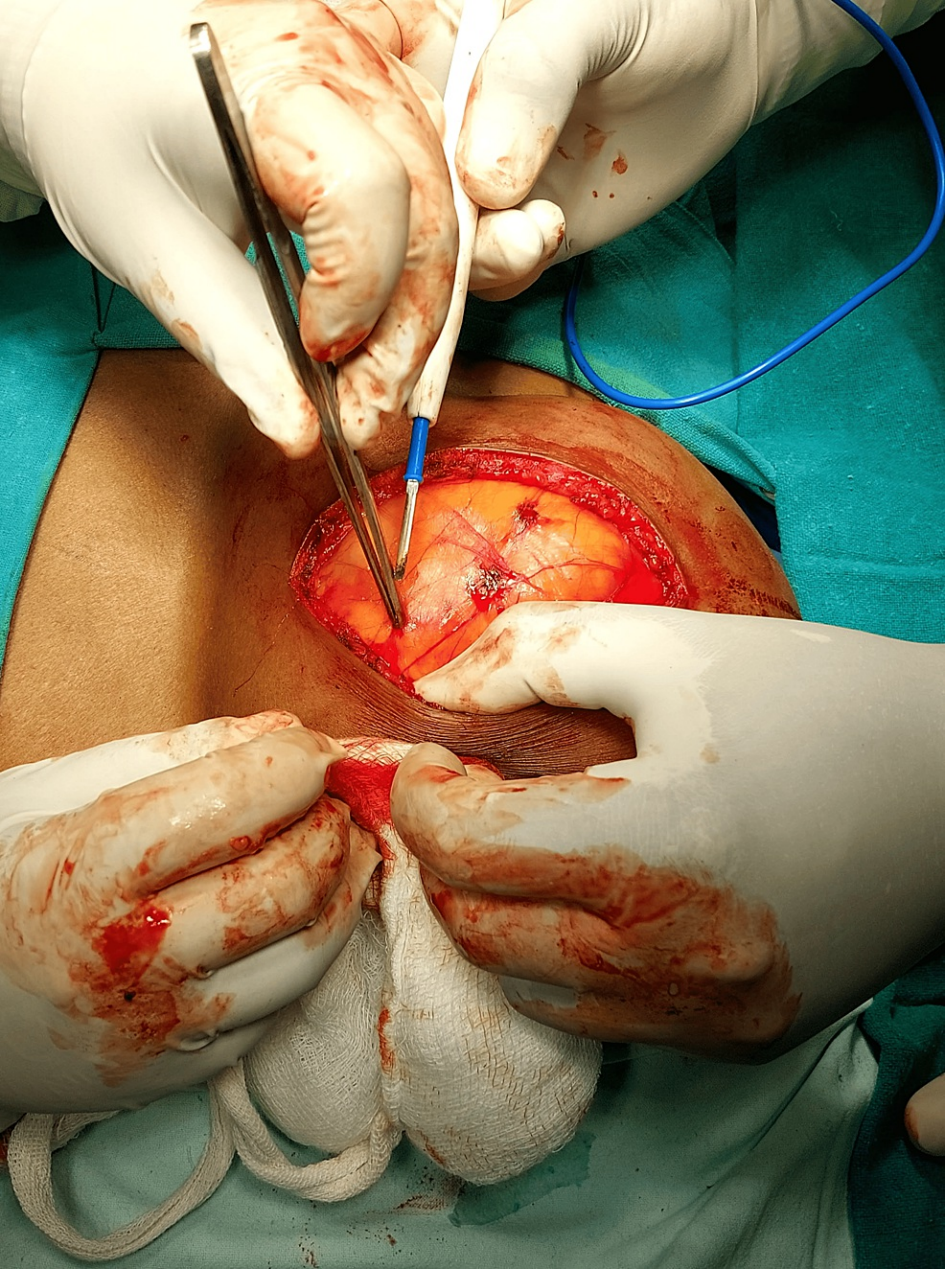
Intraoperative image An elliptical incision was made on the skin through which the lipoma was removed.

The ultrasonography (USG) of the swelling revealed an encapsulated mass of size 15 cm x 18 cm x 10 cm in the subcutaneous plane on the upper back. There was no calcification and it had minimal to no vascularity on the Doppler. Fine Needle Aspiration Cytology (FNAC) of the mass demonstrated adipocytes with large uni-vacuolated cytoplasm and dark, eccentric nuclei consistent with the clinical diagnosis of lipoma. There was no cellular atypia or malignancy. The case was managed by carrying out a complete excision of the lipoma. An elliptical incision was made on the skin, and the tumour was completely shelled out. Once the majority of the tumour was released from the fascia, the tumour was separated from underlying tissues by sharp dissection. The feeding vessel in the pedicle was carefully tied with linen and cauterised during the surgery. The tumour was removed in one piece. A liberal use of electrocautery ensured hemostasis (Figure 2).

**Figure 3:**
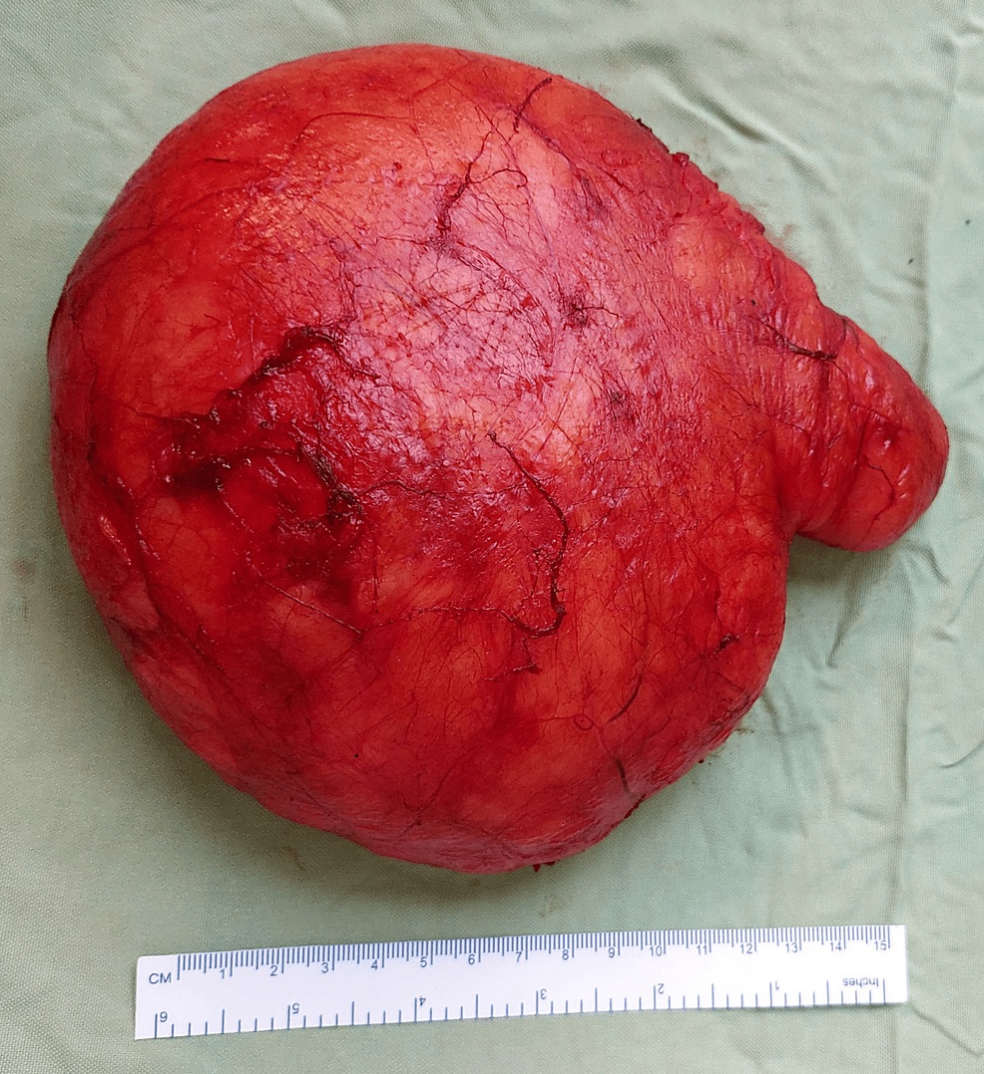
The excised specimen A 15 cm scale is kept for reference.

A suction drain with negative pressure was inserted in the postoperative dead space. The procedure was uneventful and the patient was shifted back to the ward. The histopathology of the specimen measuring 15 cm x 18 cm x 10 cm revealed uniform mature adipocytes which were regular in size with vacuolated scanty peripheral nuclei and a few capillaries confirming its benign nature (Figure 3).

## Discussion

A solitary lipoma is one of the most common soft tissue benign tumours, likely to be present in the age group 40-60 years. It is more commonly seen in females than males [3,4]. They usually have a diameter of about 2 cm and very rarely grow beyond 10 cm [5]. Theoretically, a giant lipoma is defined as a mesenchymal soft tissue tumour weighing more than 1 kg or a diameter of more than 10 cm [3-6].

Statistically, lipomas are generally smaller than 5 cm in 80% of cases, with only 1% of lesions greater than 10 cm in size [7]. Lipomas are generally only seen as cosmetic or physical deformities therefore are usually neglected by the patients. Due to their size and weight, they could potentially result in functional limitations or show signs of compression, albeit this is uncommon. Neuromuscular dysfunction can rarely be brought on by a lipoma next to an extremity's motor nerve [8-10]. Given the high incidence of fatty tumors and the comparatively low number of liposarcomas that have been found, it is likely that only a small proportion of lipomas might undergo malignant transformation [11]. Johnson et al. recommended that until proven otherwise, every soft tissue tumour measuring more than 5 cm should be deemed malignant [12].

In our case, because the tumour was more than 10 cm, an MRI and an FNAC were advised. However, since the patient was non affording, she did not undergo an MRI. An FNAC was carried out to confirm its benign nature. FNAC is a cost-effective and quick method to diagnose lipoma with high precision (sensitivity 96% and specificity 98%) [13]. The routine imaging technique for lipoma is USG. Magnetic resonance Imaging (MRI) can also be done specially to rule out malignancy (liposarcoma). Since the relative incidence of liposarcoma is very low (2.5 per million per year), an MRI should be used only when there is a strong clinical suspicion suggested by a large size, rapid growth, pain, and immobility [14]. Lipomas are traditionally managed by a complete surgical excision. Recently, liposuction and suction-assisted lipectomy have also been proposed as a viable alternative therapy for giant lipomas with a cosmetic advantage. The possible drawbacks of this procedure are limitations to visualising the tumour, fragmentation of specimen confounding the histopathology, and incomplete resection increasing chances of recurrence [15,16]. After good surgical management by any of these methods, a recurrence of lipoma is practically unheard of [16].

## Conclusions

Giant lipomas are extremely uncommon entities and contribute to only 1% of all cases of lipomas. Such a large size (more than 10 cm) always involves a possibility of malignancy. The malignancy can be suspected due to clinical features like large size, rapid growth, pain, and immobility. Imaging techniques like USG, MRI, and cytopathology should be used to confirm diagnosis and rule out malignancy. Lipoma is traditionally managed by a complete surgical excision. Recently, liposuction and suction-assisted excision have been carried out as alternative therapies. Either of these techniques, in good hands, gives permanent remedy without recurrence.

## References

[1] Mello DF, Manica MZ, Helene Júnior A (2015). Giant lipomas: a 14-case series. Rev Bras Cir Plást.

[2] Charifa A, Azmat CE; Badri T (2022). Lipoma Pathology. https://www.ncbi.nlm.nih.gov/books/NBK482343/.

[3] Silistreli OK, Durmuş EU, Ulusal BG, Oztan Y, Görgü M (2005). What should be the treatment modality in giant cutaneous lipomas? Review of the literature and report of 4 cases. Br J Plast Surg.

[4] Verdin V, Preud'Homme L, Lemaire V, Jacquemin D (2009). Giant lipoma on the back (Article in French). Rev Med Liege.

[5] Jain AK, Viswanath S (2014). Giant lipoma in the cervico-shoulder region. Int J Adv Med.

[6] Mazzocchi M, Onesti MG, Pasquini P, La Porta R, Innocenzi D, Scuderi N (2006). Giant fibrolipoma in the leg--a case report. Anticancer Res.

[7] Shin YS, Kim YJ, Park IS (2016). Sonographic differentiation between angiolipomas and superficial lipomas. J Ultrasound Med.

[8] Parihar D, Rajkawar P, Batra A, Kaur J (2013). Giant lipoma back mimicking as lipomeningomylocele-a case report. International Journal of Recent Trends in Science And Technology.

[9] Grimaldi L, Cuomo R, Castagna A, Sisti A, Nisi G, Brandi C, D'Aniello C (2015). Giant lipoma of the back. Indian J Plast Surg.

[10] Guler O, Mutlu S, Mahirogulları M (2015). Giant lipoma of the back affecting quality of life. Ann Med Surg (Lond).

[11] Sawyer KC, Sawyer RB, Lubchenco AE, Bramley HF, Fenton WC (1968). The unpredictable fatty tumor. Arch Surg.

[12] Johnson CJ, Pynsent PB, Grimer RJ (2001). Clinical features of soft tissue sarcomas. Ann R Coll Surg Engl.

[13] Khalbuss WE, Teot LA, Monaco SE (2010). Diagnostic accuracy and limitations of fine-needle aspiration cytology of bone and soft tissue lesions: a review of 1114 cases with cytological-histological correlation. Cancer Cytopathol.

[14] Niknejad M, Gaillard F (2024). Lipoma. Radiopaedia.org.

[15] Peev I, Spasevska L, Mirchevska E, Tudzarova-Gjorgova S (2017). Liposuction assisted lipoma removal - option or alternative?. Open Access Maced J Med Sci.

[16] Copeland-Halperin LR, Pimpinella V, Copeland M (2015). Combined liposuction and excision of lipomas: long-term evaluation of a large sample of patients. Plast Surg Int.

